# Selection and Validation of Reference Genes for qRT-PCR Analysis in *Neocinnamomum caudatum*

**DOI:** 10.3390/plants15131950

**Published:** 2026-06-24

**Authors:** Yi Gan, Haoyang Geng, Yuanlin Zhang, Sixin Ye, Yue Pei, Kangqi Chen, Yueping Zheng, Zhifu Zheng, Yihua Zhan

**Affiliations:** College of Advanced Agricultural Sciences, Zhejiang A&F University, Hangzhou 311300, China; ghy741858672@163.com (H.G.); senlinpengyou@stu.zafu.edu.cn (Y.Z.); 2022601022033@stu.zafu.edu.cn (S.Y.); 2023601022023@stu.zafu.edu.cn (Y.P.); 2023601021002@stu.zafu.edu.cn (K.C.); zhengyp@zafu.edu.cn (Y.Z.); zzheng@zafu.edu.cn (Z.Z.)

**Keywords:** *Neocinnamomum caudatum*, reference gene selection, quantitative RT-PCR, geNorm, NormFinder, BestKeeper, RankAggreg, Lauraceae family

## Abstract

*Neocinnamomum caudatum* (Nees) Merr. is an underutilized woody oil plant with seeds rich in long-chain fatty acids and polyunsaturated fatty acids. Reliable quantitative real-time PCR (RT-qPCR) analysis is essential for investigating the molecular mechanisms underlying seed oil biosynthesis, but suitable reference genes have not yet been validated in this species. Here, seven candidate reference genes, namely *EF-1α*, *ACT2*, *ACT11*, *UBQ11*, *TUA*, *F-BOX*, and *GAPDH*, were selected from transcriptomic data and evaluated in leaves, flowers, and developing seeds of *N. caudatum*. Their expression stability was assessed using geNorm, NormFinder, and BestKeeper, followed by comprehensive ranking with RankAggreg. Among all tested samples (leaves, flowers and developing seeds combined), *GAPDH* was identified as the most stable reference gene, whereas *EF-1α* was the least stable. For developing seeds alone, *TUA* showed the highest stability, while *EF-1α* exhibited poor stability. In leaf and flower samples, *ACT11* was the most stable gene, whereas *TUA* was unsuitable for normalization. The expression patterns of *NcFAD2* and *NcFatB*, two genes involved in fatty acid biosynthesis, were used to validate the selected reference genes. Stable reference genes and the optimized multi-gene combination generated consistent expression profiles, while unstable reference genes caused evident distortion. This study provides the first systematic evaluation of reference genes for qRT-PCR analysis in *N. caudatum* and offers a practical foundation for future functional studies of lipid metabolism in this woody oil plant.

## 1. Introduction

Woody oil tree species are important renewable resources for forestry-based bioenergy, industrial lipids, and diversified forest economies, because plant triacylglycerols represent energy-rich substitutes for fossil diesel upon conversion to fatty acid esters [1]. *Neocinnamomum caudatum* (Nees) Merr., an evergreen Lauraceae tree distributed in subtropical South China and adjacent tropical Asian regions, is a promising but underexploited woody oil resource whose seeds are rich in long-chain fatty acids, making it a potential feedstock for biodiesel and industrial lipids [2]. The genus *Neocinnamomum* is a phylogenetically distinct and monophyletic group within the Lauraceae family and comprises only a small number of tropical Asian species [3]. In addition to the ethnomedicinal value of its bark and leaves, recent phytochemical and pharmacological studies have shown that *N. caudatum* essential oils possess anti-inflammatory and antioxidant activities [4]. The major economic significance of *N. caudatum*, however, lies in its seed oil: mature seeds can contain up to 57.4% storage lipid on a dry-weight basis, with linoleic acid (35.0%), stearic acid (21.2%), oleic acid (15.8%), linolenic acid (13.1%), and palmitic acid (11.3%) as the dominant fatty acids [2]. This unusual coexistence of high stearic acid and high polyunsaturated fatty acids (PUFAs) is rare among Lauraceae species and makes *N. caudatum* a useful system for studying seed oil biosynthesis and fatty acid composition [2]. Although a developing-seed transcriptome was reported in 2018 and yielded 239,703 unigenes and 137 candidate biomarkers related to fatty acid and triacylglycerol biosynthesis [2], the temporal and tissue-specific expression patterns of many lipid-related genes in *N. caudatum* remain insufficiently characterized. In related Lauraceae species, reference gene usage has been reported in a few expression studies. For example, the ubiquitin-conjugating enzyme E2 (*UBC*) gene was employed as an internal control for RT-qPCR in *Litsea cubeba* [5,6]. However, these studies did not perform a systematic evaluation of multiple candidate reference genes, nor did they validate the stability of *UBC* across different tissues or developmental stages. To date, no systematic evaluation of reference genes has been performed in *N. caudatum*. Quantitative real-time PCR (RT-qPCR) remains a core method for relative gene expression analysis, commonly using comparative Ct-based strategies, but its reliability depends on transparent assay reporting, primer performance, RNA quality, and appropriate normalization [7,8,9,10]. The MIQE guidelines and subsequent MIQE 2.0 update emphasize that qPCR experiments must report sample handling, primer specificity, amplification efficiency, and data-analysis assumptions in sufficient detail for reproducibility [7,8]. A central requirement for reliable RT-qPCR is the use of stably expressed reference genes; however, studies using geNorm and plant-wide reference-gene validation have shown that conventional housekeeping genes are not universally stable and that single unvalidated genes can introduce large normalization errors [11,12,13,14,15]. Therefore, reference genes must be empirically validated for each species, tissue set, developmental stage, and experimental condition before downstream transcriptional analysis is interpreted [11,15]. We hypothesized that the expression stability of candidate housekeeping genes differs among tissues and developmental stages of *N. caudatum*. To address this need, the present study identifies seven candidate reference genes (*EF-1α*, *ACT2*, *ACT11*, *UBQ11*, *TUA*, *F-BOX*, and *GAPDH*) from *N. caudatum* seed transcriptome data [2]. Their expression stability across leaves, flowers, and developing seeds was evaluated using geNorm, NormFinder, and BestKeeper [11,12,13], and discrepancies among algorithms were integrated with RankAggreg [16]. Finally, *NcFatB* and *NcFAD2* were selected for validation because FatB-type acyl-ACP thioesterases influence the export of saturated fatty acids from plastids, whereas FAD2 catalyzes oleic acid-to-linoleic acid desaturation and is closely associated with seed oil composition [17,18,19,20]. This study provides the first reference-gene benchmarking framework for accurate RT-qPCR normalization in *N. caudatum* and offers a technical basis for future functional genomics and molecular breeding of this woody oil tree species.

## 2. Results

### 2.1. PCR Specificity, Amplification Efficiency & Cq Value Distribution of Seven Candidate Reference Genes

**Figure 1:**
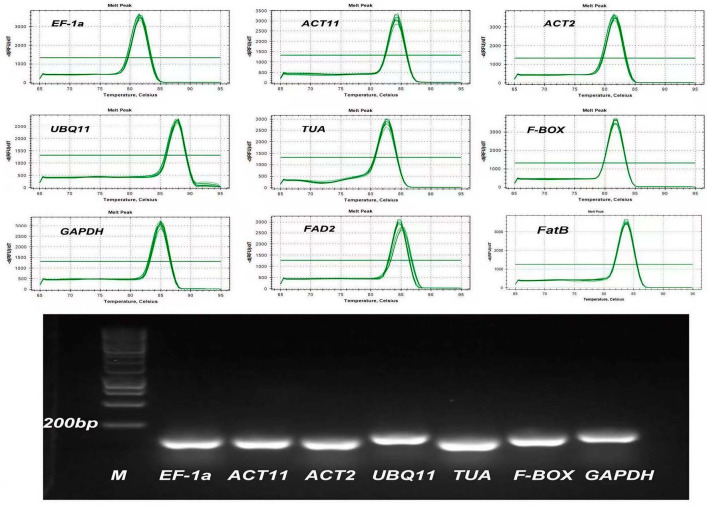
Validation of primer specificity for RT-qPCR amplification. Dissociation curves with single peaks were generated for the seven candidate reference genes and two target genes (**upper panel**). The PCR products of each primer pair showed a single band of the expected size (as listed in Table 2) on the 2% agarose gel after electrophoresis (**lower panel**). M: DL 1000 marker.

In this study, seven candidate reference genes and two target genes were identified through homology search from the *N. caudatum* transcriptome according to reference genes and lipid-metabolism genes reported in *Arabidopsis*, *N. caudatum*, and other plant systems [2,14,17,18,19,20]. The identities of the sequences to their Arabidopsis homologs ranged from 69% to 84% (Table 1). The primers for RT-qPCR were designed according to the unigene sequences with product sizes of 134–206 bp (Table 2). According to MIQE recommendations, primer specificity and amplification characteristics were checked before expression stability analysis [7,8]. After RT-qPCR experiments with pooled cDNA, every primer pair showed a single major peak in the melting curve analysis (Figure 1). Meanwhile, a single clear band with the expected size was detected in each lane of the 2% agarose gel after electrophoresis (Figure 1). The amplification efficiencies of the primers were calculated from the slopes of the standard curves [10]. They varied from 90.62% to 100.12% (Table 2). The primers of UBQ11 had the highest efficiency (100.12%), while *FatB* had the lowest efficiency (90.62%). In addition, the efficiency curves for the seven candidate reference genes had linear correlation coefficients (R^2^) ranging from 0.995 to 0.999 (Table 2, Supplemental Data S2). All primer efficiencies fell within the 90–110% range recommended by the MIQE guidelines [8].

**Figure 2:**
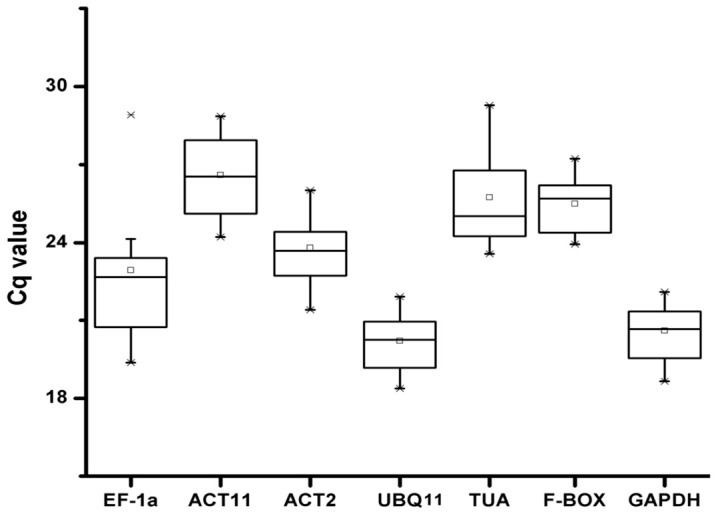
Cq distribution of seven candidate reference genes. The raw Cq of seven candidate genes across all tested samples were presented with box and whiskers plot.

To determine the expression profiles of the candidate reference genes, their average Cq values across all tissue samples (including leaf, flower and six stages of developing seeds) were demonstrated with box and whiskers plot (Figure 2). The results showed that the Cq values varied, which had a range of variation from 18.38 to 29.28. *UBQ11* had the lowest average Cq value (20.21), followed by *GAPDH* (20.59) and *EF-1α* (22.92). In contrast, *ACT11* showed the highest average Cq value (26.59) across all tissue samples. Notably, we found that most candidate reference genes had similar mean and median Cq values except for *TUA*. In addition, the outlier of *EF-1α* was far from its whiskers (Figure 2). Such variations were also confirmed by the transcriptomic data. The values of Fragments Per Kilo-base of exon per Million fragments mapped (FPKM) from seven candidate reference genes varied remarkably. *TUA*, *UBQ11* and *GAPDH* had much higher FPKM values in all sequenced seed samples than that of EF-1α and F-BOX (see Supplemental Data S1). However, the FPKM values of each reference gene displayed less variation across different seed developmental stages (S2, S4 & S6).

### 2.2. Ranking of Candidate Reference Genes by geNorm, NormFinder and BestKeeper Algorithms

**Figure 3:**
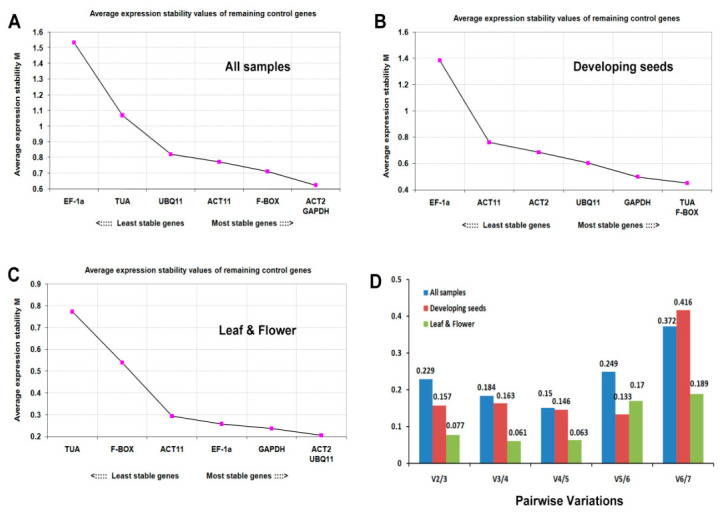
geNorm analysis of seven candidate reference genes in all tested samples (**A**), the developing seeds (**B**) and the Leaf & Flower samples (**C**). The optimal number of reference genes in each group was determined by the Pairwise variation (Vn/Vn + 1) with a cut-off value of 0.15 (**D**). The horizontal axis from left to right indicates increasing expression stability, with the least stable genes on the left and the most stable genes on the right.

**Figure 4:**
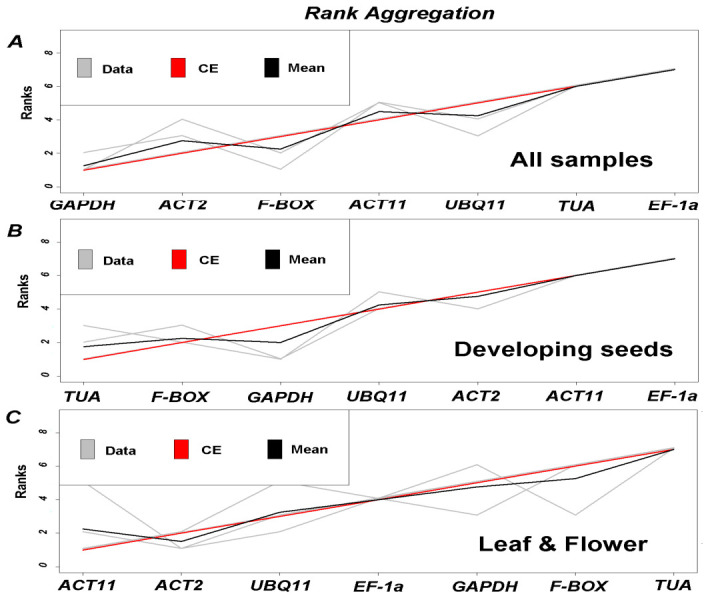
Rank aggregation of reference gene lists using the Monte Carlo algorithm: (**A**) overall sample group, (**B**) developing seeds group, (**C**) Leaf & Flower group. The solution of the rank aggregation is shown in a plot in which genes are ordered based on their rank position according to each stability measurement (gray lines). The mean rank position of each gene is shown in black, as well as the model computed by the Monte Carlo algorithm (red line).

In this study, three different algorithms, geNorm, NormFinder and BestKeeper, were used to analyze the expression stability of seven candidate reference genes [11,12,13]. In the geNorm analysis, the expression stability of a reference gene was measured with the M value, and lower M values indicate higher expression stability [11]. In addition, the pairwise variation value of Vn/Vn + 1 determines the optimal number of reference genes for accurate normalization. A cut-off value of Vn/Vn + 1 < 0.15 indicates that reference genes are sufficient and that adding another reference gene is not required for accurate normalization [11]. As shown in Figure 3A, in the overall sample group, *GAPDH* and *ACT2* showed the lowest M value, followed by *F-BOX* and *ACT11*. In contrast, the least stable gene was *EF-1α* (Figure 4A). Meanwhile, the pairwise variation V4/V5 was below the recommended 0.15 threshold, suggesting that four reference genes were sufficient for normalization under this sample set (Figure 3D). In the developing seeds group, the most stable reference genes were *TUA* and *F-BOX*, and the least stable gene was *EF-1α*. The pairwise variation value of V4/V5 was 0.146, indicating that *TUA*, *F-BOX*, *GAPDH* and *UBQ11* were required for normalization (Figure 3D). Finally, in the Leaf & Flower group, the most stable reference genes were *ACT2* and *UBQ11*, whereas the least stable reference gene was *TUA* (Figure 3C). In addition, the pairwise variation value of V2/V3 was 0.077, showing that *ACT2* and *UBQ11* were sufficient for normalization.

In the NormFinder analysis, *GAPDH* was ranked as the most stable reference gene both in the overall samples group (Stability Value = 0.285) and the developing seeds group (SV = 0.254). In contrast, *ACT2* was listed as the most stable reference gene in the Leaf & Flower group (SV = 0.247, Table 3).

The Excel-based BestKeeper algorithm is often used to evaluate expression stability of reference genes based on the coefficient of variance (CV) and the standard deviation (SD) of the average Cq values [13]. In the present study, the candidate reference genes were ranked according to their SD values. As shown in Table 4, *GAPDH* had the lowest SD value in both the overall samples group (SD = 0.77) and the developing seeds group (SD = 0.85). In contrast, *EF-1α* was the least stable reference gene in both groups. This result was consistent with the output of NormFinder (Table 3). In the Leaf & Flower group, *ACT11* had less variation than the other six candidate genes (SD = 0.26, Table 4). However, *F-BOX* in the overall sample group and *TUA* in the developing seeds group had the lowest CV values instead of *GAPDH* (Table 4), indicating that the ranking output by CV and SD in BestKeeper requires further comprehensive analysis.

To generate a comprehensive final ranking of the candidate reference genes ranked by three algorithms, we used RankAggreg, an R package for weighted rank aggregation [16]. As shown in Figure 4, *GAPDH* was ranked as the most stable gene for all samples, followed by *ACT2*, while *EF-1α* was the least stable reference gene. In the developing seeds group, *TUA*, instead of *GAPDH*, was ranked as the best reference gene, and *EF-1α* was still the least stable gene. Finally, in the Leaf & Flower group, *ACT11* was listed as the best reference gene and *TUA* was the least stable gene.

### 2.3. Expression Analysis of NcFatB and NcFAD2 Genes for Reference Gene Validation

**Figure 5:**
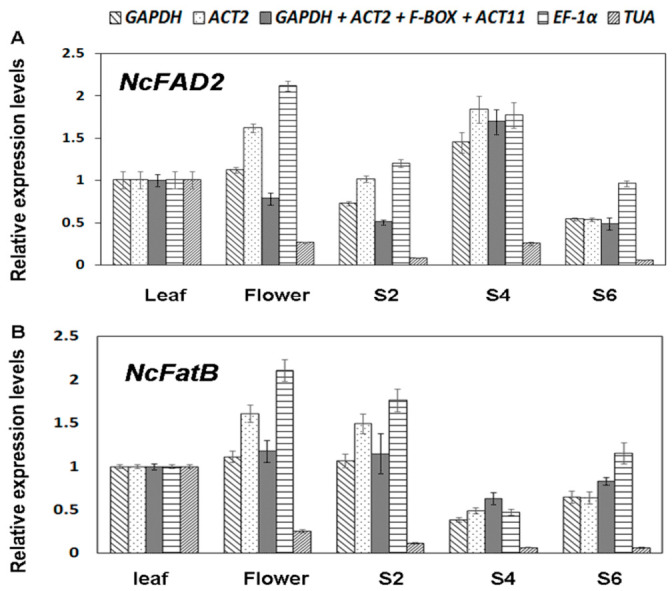
Validation of reference genes with RT-qPCR. The relative expression levels of *Fatty Acid Desaturase 2* (*FAD2*, (**A**)) and *Acyl-thioesterase B* (*FatB*, (**B**)) in the Leaf, flower and the developing seed samples (52, 96 and 146 DAF, marked as S2, S4 & S6, respectively) were normalized with both individual (*GAPDH*, *ACT2*, *TUA* & *EF-1α*) and a combination of reference genes (*GAPDH*, *ACT2*, *F-BOX* & *ACT11*). To clearly show the expression changes during the key stages of oil accumulation, only the data for S2, S4, and S6 are shown; the complete data for S1, S3, and S5 are provided in Supplemental Data S4.

To validate the accuracy of the reference genes, the *FATTY ACID DESATURASE 2* (*FAD2*) and *Acyl-ACP thioesterase B* (*FatB*) genes from *N. caudatum* were selected (Table 1), because *FAD2* is essential for polyunsaturated lipid synthesis and FatB-type acyl-ACP thioesterases affect the amount and type of saturated fatty acids exported from plastids [17,18]. Their expression levels in leaves, flowers and developing seeds (52, 96 and 146 DAF, marked as S2, S4 and S6, respectively) were quantified and normalized with both single and combined reference genes. In the geNorm analysis, the combination of *GAPDH*, *ACT2*, *F-BOX* and *ACT11* was listed as the optimal reference gene combination for gene normalization in overall samples (Figure 3A,D). As shown in Figure 5A, when normalized with the combination of four reference genes, *NcFAD2* increased to its peak expression level at the S4 stage of seeds and then decreased to the lowest level at the S6 stage. Normalization with a single reference gene, *GAPDH* or *ACT2*, resulted in similar expression patterns, although the fold changes in *NcFAD2* expression were slightly different (Figure 5A). In contrast, normalization with *EF-1α* or *TUA*, which were ranked as unstable reference genes by RankAggreg (Figure 4), led to evident distortion of the *NcFAD2* expression pattern. The peak expression level of *NcFAD2* was presented in flowers instead of S4 seeds when normalized with *EF-1α*, while the lowest expression level was still presented in S6 seeds. Similarly, normalization with *TUA* resulted in a sharp decrease in *NcFAD2* expression in flowers and developing seeds, and the highest expression level of *NcFAD2* was presented in leaves rather than developing seeds (Figure 5A). Similar results were observed for *NcFatB.* When using the most stable reference gene *GAPDH* or the four-gene combination (*GAPDH*, *ACT2*, *F-BOX* and *ACT11*) for gene normalization, the highest expression level of *NcFatB* was presented in flowers, followed by S2 seeds. Then, the transcription abundance of *NcFatB* declined sharply in S4 seeds and increased slightly in S6 seeds (Figure 5B). In contrast, normalization with *EF-1α* led to more obvious fluctuation of *NcFatB* expression in all tested samples. Furthermore, when *TUA* was used as the internal standard, an unexpected decline of *NcFatB* expression occurred in flower, S2, S4 and S6 seed samples (Figure 5B). Taken together, the expression patterns of *NcFatB* and *NcFAD2* were highly dependent on the stability of the reference genes. Normalization with *GAPDH* and the four-gene combination resulted in more reliable gene expression estimates for all tissue samples of *N. caudatum*.

## 3. Discussion

Precise transcriptional quantification by RT-qPCR is crucial for dissecting metabolic networks and functional genomics in forest trees, but it is technically reliable only when assay quality and normalization strategy are rigorously controlled [7,8,21]. Because no universally stable reference gene exists across all plant species, tissues, developmental stages, and environmental conditions, empirical screening of internal controls remains a fundamental requirement in plant molecular biology [11,14,15]. The present study addresses this requirement by establishing the first comprehensive reference gene verification framework for *N. caudatum*, a non-model woody oil tree with high economic potential. Our integrated analysis using geNorm, NormFinder, and BestKeeper revealed a clear tissue-specific pattern of expression stability: *GAPDH* was the most stable gene across all combined vegetative and re-productive samples; *TUA* exhibited superior stability within developing seeds; whereas *EF-1α*, a conventional housekeeping gene frequently used in plant expression studies, showed poor stability in the overall and developing-seed groups [14,22].

The high stability of *GAPDH* in the overall sample set is consistent with findings in related or economically important woody plants. For example, *GAPDH* was validated as the optimal internal control for different tissues of *Cinnamomum cassia* and *C. cassia* var. macrophyllum [23], and *GAPDH* also performed well under specific conditions in *Eucommia ulmoides* and Chinese fir [21,24]. However, this does not mean that *GAPDH* is universally suitable; studies in moso bamboo and other plants have shown that the most stable reference genes differ substantially among tissues and developmental stages [25]. Conversely, the instability of *EF-1α* in *N. caudatum* contrasts with studies in *Rhizophora apiculata* and *Cinnamomum burmannii*, where *EF1α* was recommended under particular tissue or experimental groupings [22,26]. One possible explanation is that *EF-1α* expression is highly sensitive to developmental transitions and metabolic activity in seeds and flowers. In many plants, elongation factor genes are involved in protein synthesis and can be transcriptionally regulated by growth stage, nutrient status, and hormonal signals. The wide variation in Cq values of *EF-1α* across our samples (Figure 2) and its high coefficient of variation (CV) and standard deviation (SD) in BestKeeper analysis (Table 4) support this view. However, further targeted experiments are needed to identify the exact regulatory factors affecting *EF-1α* stability in *N. caudatum*. These cross-species differences reinforce the principle that reference gene stability is condition-specific rather than strictly conserved by phylogenetic relationship [11,15]. Thus, internal controls should not be transferred from related taxa to *N. caudatum* without de novo validation. Notably, even within the Lauraceae family, reference gene stability can differ between species. For instance, while *UBC* was used as a reference in *Litsea cubeba* without systematic validation [5,6], our study demonstrates that *UBQ11* (a ubiquitin family gene) is not always the most stable across all sample sets in *N. caudatum* (Table 3 and Table 4, Figure 4). This further emphasizes the need for species-specific reference gene validation.

Beyond statistical ranking, a useful reference-gene framework should preserve biologically plausible expression patterns of target genes linked to agronomic traits such as seed oil accumulation [11,22]. Fatty acid composition in oil plants is influenced by coordinated expression of enzymes such as *FatB* and *FAD2*: FatB-type acyl-ACP thioesterases affect the amount and type of fatty acids exported from plastids, while *FAD2* catalyzes oleic acid-to-linoleic acid desaturation and contributes to seed fatty acid profiles [17,18,19,20]. When normalized using *GAPDH* or the optimized multi-gene combination (*GAPDH + ACT2 + F-BOX + ACT11*), *NcFAD2* and *NcFatB* showed coherent developmental expression patterns across leaves, flowers, and developing seeds. By contrast, normalization with the unstable genes *EF-1α* or *TUA* markedly altered the apparent expression trends, demonstrating that the choice of internal control directly affects biological interpretation. These results are consistent with previous evidence that carefully selected multiple reference genes can improve RT-qPCR normalization and reduce the risk of false expression patterns [11].

The distortion observed when *EF-1α* or *TUA* was used as a single internal control is not a trivial technical issue. In our validation analysis, unstable normalization changed the apparent tissue or developmental stage at which *NcFAD2* and *NcFatB* reached maximum abundance, which could lead to erroneous hypotheses about lipid-biosynthetic regulation. Similar biases caused by unsuitable reference genes have been reported in *Rhizophora apiculata* and *Eucommia ulmoides*, where target gene expression patterns changed substantially depending on the internal control used [24,26]. Therefore, the present results provide practical evidence that an optimized reference framework is a prerequisite for accurate investigation of fatty acid and PUFA biosynthesis in *N. caudatum*.

Despite these findings, several limitations of this study should be acknowledged. First, the reference gene stability analysis was performed only under normal physiological conditions and was limited to leaves, flowers, and developing seeds of *N. caudatum*. Therefore, the recommended reference genes may not be directly applicable to other organs, environmental stresses, hormone treatments, pathogen challenges, or different genetic backgrounds without further validation [21,27,28]. Second, the seven candidate reference genes were selected mainly from available seed transcriptome resources, which may restrict the diversity of potential internal controls. Inclusion of additional candidate genes, such as PP2A, TIP41, SAND, ribosomal protein genes, or other stably expressed genes identified from broader transcriptomic datasets, may further improve the robustness of reference gene selection. Third, the validation of reference genes was conducted using only two lipid biosynthesis-related genes, *NcFAD2* and *NcFatB*. Although these genes are relevant to seed oil metabolism, additional target genes involved in different biological pathways should be tested in future studies. Finally, the plant materials were collected from a limited number of individuals at a single location, and broader sampling across populations, developmental years, and ecological conditions would help verify the general applicability of the proposed normalization strategy. Thus, the reference genes recommended in this study should be considered reliable for the specific tissues and developmental stages examined here, while further validation is necessary before their use under other experimental conditions. In particular, because *TUA* proved most stable in developing seeds, the key tissue for oil accumulation, future studies on the temporal dynamics of fatty acid biosynthetic genes during seed maturation should prioritize *TUA* as the normalization control. Moreover, the instability of *EF-1α* observed here suggests that this commonly used gene should be avoided in *N. caudatum* without prior validation, and future research extending to abiotic stress or hormone treatments should include a fresh stability screening using the same multi-algorithm pipeline.

In conclusion, this study represents a major step forward for *N. caudatum* functional genomics by identifying *GAPDH* and *TUA* as suitable reference genes for the tested tissue/developmental contexts in *N. caudatum.* By integrating multi-algorithmic evaluations with robust rank aggregation, we have eliminated the technical ambiguity that often plagues gene expression studies in non-model forest trees. This optimized framework not only guarantees high-fidelity quantification of pathways regulating fatty acid and PUFA biosynthesis but also provides a powerful technological springboard for subsequent molecular marker-assisted selection, metabolic engineering, and genetic improvement programs aimed at maximizing the potential of this valuable woody oil resource.

## 4. Materials and Methods

### 4.1. Plant Material and Tissue Sampling

From August 2015 to February 2016, 100 g of mature leaves, flowers and developing seeds (harvested at 20, 52, 81, 96, 126 and 146 days after flowering, corresponding to stages S1 through S6, respectively, Figure S1) were collected. For each tissue and developmental stage, samples were collected independently from three individual trees at Xishuangbanna Tropical Botanical Garden, Yunnan Province, China (E 101.251517°, N 21.936948°) and treated as three biological replicates. The samples were immediately flash-frozen in liquid nitrogen and stored at –80 °C for subsequent RNA extraction. RNA extraction, cDNA synthesis and qRT-PCR were performed independently for each biological replicate within a few days of sampling.

### 4.2. RNA Extraction and cDNA Synthesis

Total RNA was extracted using the RNeasy Plant Mini Kit (Cat No. 74904, Qiagen, Germantown, MD, USA) and treated with DNase I (Cat No. M6101, Promega Corporation, Madison, WI, USA) to eliminate residual genomic DNA. RNA integrity was assessed by 1% agarose gel electrophoresis (Figure S2), which revealed intact 28S and 18S rRNA bands without visible degradation. RNA concentration and purity were determined using a Nanodrop ND-2000 spectrophotometer (Nanodrop Technologies, Wilmington, DE, USA; software v1.6); all samples exhibited an A260/A280 ratio between 1.8 and 2.1. First-strand cDNA was synthesized from 1 µg of total RNA using the SuperScript™ III First-Strand Synthesis System (Cat No. 18080-051, MAN0001346 Rev. 3.0, Invitrogen, Carlsbad, CA, USA) according to the manufacturer’s protocol.

### 4.3. Identification of Candidate Reference Genes from RNA-Seq Data and Gene Specific Primer Design

Nine cDNA libraries of *N. caudatum* developing seeds (52, 96 and 146 days after flowering, three biological replicates) were deep-sequenced on the Illumina NextSeq 500 platform in the previous transcriptome study [2]. The raw sequencing data are available in the NCBI SRA database (https://www.ncbi.nlm.nih.gov/sra) under accession numbers SRX4402579-SRX4402587 [2]. After assembly, 239,703 unigenes were obtained [2].

Using BioEdit software (Version 1.6.0.1), local BLAST searches were performed using BLAST+ (v2.13.0) between these unigenes and seven conventional housekeeping genes, along with two target genes in Arabidopsis (*EF-1α*, *ACT2*, *ACT11*, *UBQ11*, *TUA*, *F-BOX*, *GAPDH*, *FatB* and *FAD2*; see Table 1) [29]. For each BLAST search, the unigene with the lowest E-value was selected, and its FPKM value was verified to ensure sufficient expression abundance across different tissues (Supplemental Data S1). Open reading frames (ORFs) were predicted using Getorf (EMBOSS package, https://emboss.sourceforge.net [30]), and gene-specific primers were designed within the ORF regions using Vector NTI Advanced software (Version 11.5, Invitrogen, Carlsbad, CA, USA) with the following parameters: 18–22 bp length, amplicon size of 150–200 bp, melting temperature (Tm) around 60 °C, and GC content of 40–60%.

To further confirm orthology, the deduced amino acid sequences of these unigenes were aligned against the *Arabidopsis thaliana* proteome database via BLASTP (https://blast.ncbi.nlm.nih.gov). The initial screening of candidate genes during our transcriptomic survey was governed by the lowest E-value and highest bit score to ensure that the optimal evolutionary match was captured, rather than relying on an arbitrary lower-limit cut-off of nucleotide identity percentage. The comprehensive alignment metrics at both nucleotide and protein levels are summarized in Table 1.

### 4.4. Quantitative Real-Time PCR and Amplification Efficiency

To verify primer specificity, PCR products were resolved on a 2% agarose gel via electrophoresis. RT-qPCR assays were conducted in 96-well plates (Axygen, Union City, CA, USA, https://www.axygen.com) and performed on a Bio-Rad CFX-96 RT-qPCR system (v3.0, Bio-Rad, Hercules, CA, USA) with SYBR Premix Ex Taq dye (Cat No. RR420A, Takara Bio Inc., Shiga, , Japan). Each 20 μL reaction mixture contained 1 μL cDNA, 0.5 μL of each primer, 8 μL ddH_2_O and 10 μL SYBR Premix. The RT-qPCR amplification conditions consisted of an initial denaturation at 95 °C for 3 min, followed by 40 cycles of 95 °C for 10 s and 58 °C for 30 s, with a final melting curve analysis performed to verify amplicon specificity, following standard RT-qPCR reporting guidelines [7,8]. All RT-qPCR experiments included three biological replicates.

To determine amplification efficiency, a five-fold dilution series of pooled cDNA (1, 1/5, 1/25, 1/125, 1/625 and 1/3125) was subjected to qPCR (Supplemental Data S2). The standard curves were used to calculate slope values and coefficients of determination (R^2^). Primer efficiency (E) was calculated as E = (10^(−1/slope) − 1) × 100 [10].

### 4.5. Statistical Analysis of Expression Stability and Rank Aggregation

Three different algorithms, geNorm, NormFinder and BestKeeper, were used to analyze the stability of the reference genes [11,12,13]. For geNorm and NormFinder analyses, Cq values were converted to relative quantities prior to analysis [11,12]; for BestKeeper, raw Cq values were used directly [11]. To obtain a consensus ranking, the outputs of the three algorithms were integrated using the RankAggreg package (v0.6.5) in R (v3.6.1) [16,31]. Rank aggregation aims to find an aggregated ranking that minimizes the distance to each ranked list in the input set [16]. The distance among ordered lists was calculated using the Spearman footrule function. Four individual rankings of reference genes output by M value (geNorm), stability value (NormFinder), SD and CV (BestKeeper) were used as input. Notably, the initial M values in geNorm analysis for calculating the normalization factor value were used because the final M values of the two most stable genes were the same. The RankAggreg script is provided in Supplemental Data S5.

### 4.6. Validation of Reference Genes with RT-qPCR

To evaluate the reliability of the identified reference genes, two lipid biosynthesis-related genes from *N. caudatum*, namely *Acyl-ACP thioesterase B* (*FatB*) and *Fatty Acid Desaturase 2* (*FAD2*), were selected for validation. Their expression levels were measured in leaves, flowers, and developing seeds (stages S2, S4, and S6) and normalized using both single reference genes (*GAPDH*, *ACT2*, *TUA*, and *EF-1α*) and a combination of four reference genes (*GAPDH*, *ACT2*, *F-BOX*, and *ACT11*). The raw data and calculations are provided in Supplemental Data S4.

## 5. Conclusions

In conclusion, seven candidate reference genes were identified from *N. caudatum* transcriptomic resources and systematically evaluated across leaves, flowers and developing seeds. *GAPDH* was the most stable gene across all samples, *TUA* performed best in developing seeds, and *ACT11* showed the highest stability in the leaf and flower group based on the comprehensive RankAggreg ranking. In contrast, *EF-1α* was unstable across the overall and developing-seed groups. These validated reference genes will support accurate RT-qPCR normalization and future functional studies of fatty acid metabolism and seed oil biosynthesis in *N. caudatum* and related Lauraceae species.

## Figures and Tables

**Table 1:** Description of candidate reference genes and target genes for RT-qPCR in *N. caudatum*.

Gene	Unigene ID.	Arabidopsis Homolog Locus	Genbank Accession Number	E-Value	Protein Identity (BLASTP)	Protein Positives (BLASTP)	Description
EF-1α	c104567_g1_i1	AT5G60390	PZ506889	0	96.43%	98%	GTP binding Elongation factor Tu family protein
ACT11	c108311_g4_i1	AT3G12110	PZ506888	0	97.35%	99%	Actin11
ACT2	c108311_g1_i1	AT3G18780	PZ506887	1 × 10^−141^	97.88%	98%	Actin 2
UBQ11	c112907_g2_i2	AT4G05050	PZ506897	2 × 10^−88^	100%	100%	Ubiquitin11
TUA	c117536_g9_i2	AT4G14960	PZ506895	0	97.56%	98%	Alpha-6 Tubulin
F-BOX	c101252_g1_i1	AT3G53000	PZ506892	3 × 10^−8^	71.81%	83%	Phloem protein 2-A15
GAPDH	c94452_g1_i1	AT1G13440	PZ506893	1 × 10^−136^	90.66%	96%	Glyceraldehyde-3-phosphate dehydrogenase C-2
FATB	c109678_g1_i1	AT1G08510	MG763172	3 × 10^−27^	71.85%	84%	Plant acyl-thioesterase B
FAD2	c109710_g1_i1	AT3G12120	PZ506891	1 × 10^−28^	67.27%	78%	Fatty acid desaturase 2

**Table 2:** Gene-specific primers sequence for detection by RT-qPCR.

Gene	Primer Sequences (FP + RP)	Tm (°C)	Product Length (bp)	PCR Efficiency E (%)	R^2^
EF -1α	AATATTGTGGTCATTGGCCATGTCG	60.2	135	93.12	0.999
	TGATCTCTTGTTCATCTCGGCAGC				
ACT11	ATGGCAGACGGCGAGGATATTCA	60.5	137	94.22	0.999
	ATCACACCAGTGTGGCGTGGAC				
ACT2	TCCTTACTGAGGCCCCTCTTAACCC	60.3	136	95.67	0.999
	GTGGTACGGCCACTGGCATATAGAG				
UBQ11	GACTCTCACTGGTAAGACGATTAC	59.8	175	100.12	0.999
	CTCCTTCTGTATGTTGTAATCGG				
TUA	CCAAGCTTGGTTTCACTGTCTACCC	59.9	134	92.88	0.999
	GATCGCCTCATTGTCAAGCAGGA				
F-BOX	GATTCAAACTGAAGAATCCAGG	61.2	174	93.63	0.999
	GTTACAGATATGGCGTCCAAG				
GAPDH	AGATTTGGCATTGTTGAGGGTTTGA	59.5	183	92.6	0.995
	TTTTCCATTCAACGCCGGGA				
FatB	AGAAACAGTGGACGAATCTTG	60.9	206	90.62	0.998
	TCCGTACATGGTTAAGTGCA				
FAD2	TTGATCACTTACCTGCAGC	59	183	97.53	0.998
	GTAGTGGGGCATGTTAGAGAA				

**Table 3:** Ranking of reference genes based on their stability value calculated by NormFinder algorithm.

All Samples		Developing Seeds		Leaf & Flower	
Reference Gene	Stability Value	Reference Gene	Stability Value	Reference Gene	Stability Value
*GAPDH*	0.285	*GAPDH*	0.254	*ACT2*	0.247
*ACT2*	0.346	*F-BOX*	0.337	*ACT11*	0.255
*F-BOX*	0.383	*TUA*	0.383	*UBQ11*	0.293
*ACT11*	0.547	*ACT2*	0.397	*EF-1α*	0.336
*UBQ11*	0.593	*UBQ11*	0.551	*GAPDH*	0.355
*TUA*	0.755	*ACT11*	0.611	*F-BOX*	0.546
*EF-1α*	1.095	*EF-1α*	1.345	*TUA*	0.783

**Table 4:** Ranking of reference genes based on the SD values calculated by BestKeeper algorithm.

All Samples			Developing Seeds			Leaf & Flower		
RG	Std Dev	CV	RG	Std Dev	CV	RG	Std Dev	CV
*GAPDH*	0.77	3.93	*GAPDH*	0.85	4.35	*ACT11*	0.26	0.98
*F-BOX*	0.86	3.39	*TUA*	0.89	3.58	*ACT2*	0.40	1.68
*UBQ11*	0.94	4.70	*F-BOX*	0.96	3.80	*UBQ11*	0.42	2.10
*ACT2*	1.06	4.48	*UBQ11*	1.08	5.35	*EF-1a*	0.43	1.94
*ACT11*	1.33	4.98	*ACT2*	1.27	5.39	*F-BOX*	0.44	1.70
*TUA*	1.45	5.65	*ACT11*	1.68	6.33	*GAPDH*	0.60	2.85
*EF-1α*	1.75	7.65	*EF-1a*	2.12	9.30	*TUA*	0.91	3.25

## Data Availability

The raw sequencing reads of RNA-Seq are available in the Sequence Read Archive at NCBI (Accession numbers: SRX4402581, SRX4402582, SRX4402579, SRX4402580, SRX4402585, SRX4402586, SRX4402583, SRX4402584 and SRX4402587). [https://www.ncbi.nlm.nih.gov/sra/?term=neocinnamomum+caudatum] (accessed on 16 June 2026). The FPKM values of 7 candidate genes, primer-efficiency data and stability-analysis input files are provided in Supplemental Data S1–S3. The nucleotide sequences of the seven candidate reference genes (*ACT2*, *ACT11*, *EF-1α*, *F-BOX*, *GAPDH*, *TUA*, *UBQ11*) and the two target genes have been deposited in the NCBI GenBank database under accession numbers PZ506887–PZ506897. The sequence of *FatB* has been deposited under accession number MG763172. These sequences are publicly available and can be accessed via the NCBI website.

## Supplementary Materials

The following supporting information can be downloaded at https://www.mdpi.com/article/10.3390/plants15131950/s1: Supplemental Data S1: FPKM values of 7 candidate reference genes. Supplemental Data S2: Calculation of primer efficiency with standard curves. Supplemental Data S3: Raw Cq values for stability-analysis inputs. Supplemental Data S4: Validation of FAD2 & FATB gene expression levels with combination of multiple reference genes. Supplemental Data S5: The script of RankAggreg package in R program. Supplemental Figure S1: Tissue samples of *Neocinnamomum caudatum *(A) mature leaves of N. caudatum; (B) Young flowers of N. caudatum; (C) Developing seeds of N. caudatum, S1-S6 represent for 20, 52, 81, 96, 126 and 146 days after flowering, respectively. Supplemental Figure S2: RNA extracted from mature leaves(L), Young flowers(F) and Developing seeds of *N. caudatum.* S1-S6 represent for 20, 52, 81, 96, 126 and 146 days after flowering. Supplemental Figure S3: Agarose gel electrophoresis validation of primer specificity for RT-qPCR. Upper panel: Melting curves showing single peaks for each primer pair. Lower panel: Agarose gel (2%) showing single bands of expected sizes for all tested candidate reference genes and target genes. M: DL1000 DNA marker. The positions of the 200 bp marker band are indicated.

## Abbreviations

The following abbreviations are used in this manuscript:

RT-qPCR	Quantitative real-time PCR
PUFAs	Polyunsaturated fatty acids
