# Supplementary material for: Selection and Validation of Reference Genes for qRT-PCR Analysis in Neocinnamomum caudatum
Source: Plants (Basel). 2026 Jun 24;15(13):1950. doi: 10.3390/plants15131950 (PMC13363998; doi:10.3390/plants15131950)
Supplement: Supplementary file 1 [file plants-15-01950-s001.zip › Supplemental data 5.pdf]

The RankAggreg v. 0.4-3 (http://cran.r-project.org/web/packages/RankAggreg/) package of R program v. 3.0.1 (http://www.r-project.org/) was used to merge the stability measurements obtained from the three Excel-based tools and establish a consensus rank of RGs (Pihur and Datta 2009). The rankings from four terms 'Stab', 'CV', 'r2' and 'M' were used as input. The R script file example is available in Appendix S1 (see Sup-porting Information) or in page 5 of the PDF (http://cran.r-project.org/web/packages/RankAggreg/RankAggreg.pdf). According to the size of the ranking list (16) we used Monte Carlo algorithm to calculate and visually present the rank aggregation by line chart. The rank aggregation aims at finding an aggregated ranking that minimizes the distance to each of the ranked lists in the input set. The distance among ordered lists is calculated using the Spearman foot rule function. Because geNorm yielded the same M stability value in the chart of the two most stable genes, we distinguished these two genes' order by the initial M value when calculating the normalization factor (NF) value.

```
x <- matrix(c(
"G","A","B","F","C","D","E",
"G","F","B","D","A","E","C",
"B","G","A","E","C","D","F",
"A","F","G","D","B","C","E"
), byrow=TRUE, ncol=7)
 (CES <- RankAggreg(x, 7, method="CE", distance="Spearman", rho=.1,
verbose=FALSE))
plot(CES,lwd=3)
```
