# Supplementary material for: Selection and Validation of Reference Genes for qRT-PCR Analysis in Neocinnamomum caudatum
Source: Plants (Basel). 2026 Jun 24;15(13):1950. doi: 10.3390/plants15131950 (PMC13363998; doi:10.3390/plants15131950)
Supplement: Supplementary file 1 [file plants-15-01950-s001.zip › plants-4363453-supplementary.pdf]

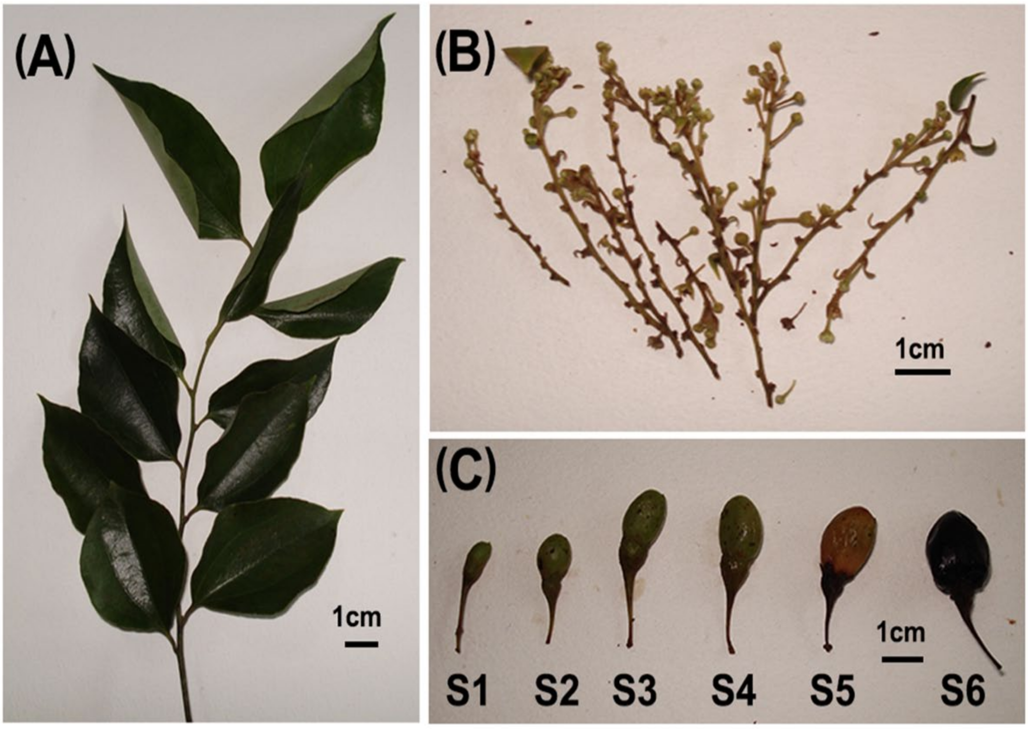

Figure S1. Tissue samples of *Neocinnamomum caudatum*. (A) mature leaves of N. caudatum; (B) Young flowers of *N. caudatum*; (C) Developing seeds of *N. caudatum*, S1-S6 represent for 20, 52, 81, 96, 126 and 146 days after flowering, respectively.

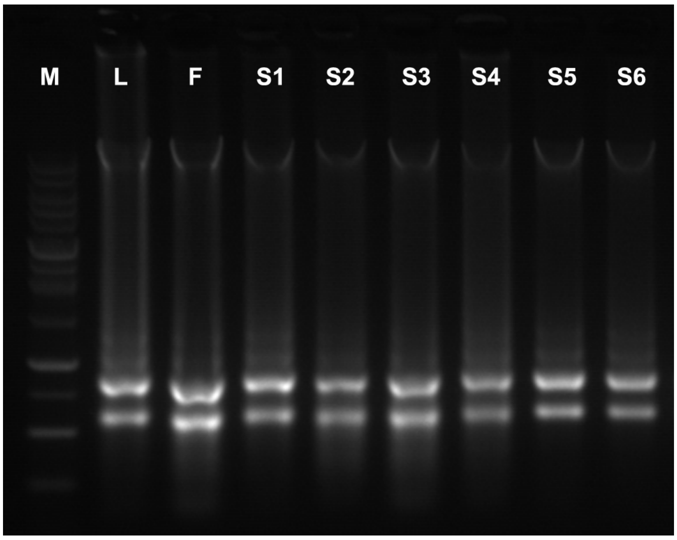

Figure S2. RNA extracted from mature leaves(L), Young flowers(F) and Developing seeds of *N. caudatum.* S1-S6 represent for 20, 52, 81, 96, 126 and 146 days after flowering.

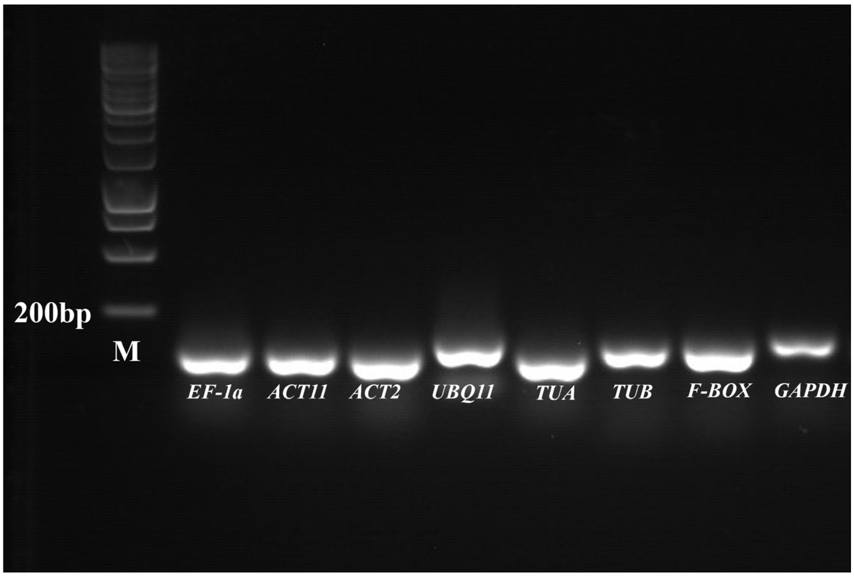

Figure S3. Agarose gel electrophoresis validation of primer specificity for RT-qPCR. Upper panel: Melting curves showing single peaks for each primer pair. Lower panel: Agarose gel (2%) showing single bands of expected sizes for all tested candidate reference genes and target genes. M: DL1000 DNA marker. The positions of the 200 bp marker band are indicated.
